# Comprehensive framework of GPU-accelerated image reconstruction for photoacoustic computed tomography

**DOI:** 10.1117/1.JBO.29.6.066006

**Published:** 2024-06-06

**Authors:** Yibing Wang, Changhui Li

**Affiliations:** a Peking University, College of Future Technology, Department of Biomedical Engineering, Beijing, China; b Peking University, National Biomedical Imaging Center, Beijing, China

**Keywords:** photoacoustic computed tomography, large-scale data size, GPU-accelerated method, Taichi Lang for Python, multiple GPU platform

## Abstract

**Significance:**

Photoacoustic computed tomography (PACT) is a promising non-invasive imaging technique for both life science and clinical implementations. To achieve fast imaging speed, modern PACT systems have equipped arrays that have hundreds to thousands of ultrasound transducer (UST) elements, and the element number continues to increase. However, large number of UST elements with parallel data acquisition could generate a massive data size, making it very challenging to realize fast image reconstruction. Although several research groups have developed GPU-accelerated method for PACT, there lacks an explicit and feasible step-by-step description of GPU-based algorithms for various hardware platforms.

**Aim:**

In this study, we propose a comprehensive framework for developing GPU-accelerated PACT image reconstruction (GPU-accelerated photoacoustic computed tomography), to help the research community to grasp this advanced image reconstruction method.

**Approach:**

We leverage widely accessible open-source parallel computing tools, including Python multiprocessing-based parallelism, Taichi Lang for Python, CUDA, and possible other backends. We demonstrate that our framework promotes significant performance of PACT reconstruction, enabling faster analysis and real-time applications. Besides, we also described how to realize parallel computing on various hardware configurations, including multicore CPU, single GPU, and multiple GPUs platform.

**Results:**

Notably, our framework can achieve an effective rate of ∼871 times when reconstructing extremely large-scale three-dimensional PACT images on a dual-GPU platform compared to a 24-core workstation CPU. In this paper, we share example codes via GitHub.

**Conclusions:**

Our approach allows for easy adoption and adaptation by the research community, fostering implementations of PACT for both life science and medicine.

## 1 Introduction

Photoacoustic (PA) computed tomography (PACT) is a non-invasive imaging modality with various applications in life science research and clinical settings, such as cancer, vascular mapping, and therapy guiding and monitoring.^1^^,^^2^ In PACT, a short-pulsed laser illuminates the tissue, generating acoustic waves (PA signal) due to thermoelastic expansion after the tissue absorbs photon energy. These acoustic waves are detected by ultrasonic transducers (USTs) and then used to reconstruct the initial pressure distribution in the tissue, which is directly related to the spatial distribution of the optical absorption.^3^^,^^4^ PACT combines the advantages of ultrasound and optical imaging, offering high spatial resolution, deep imaging depth, and functional imaging capability.^5^^–^^8^ However, PACT imaging faces challenges in fast image reconstruction due to the massive size of data with the quick increase in the total number of UST elements.^9^^,^^10^

The image reconstruction process in PACT involves solving an inverse problem based on the detected time-resolved acoustic signals. Various image reconstruction algorithms have been developed for PACT, including time-domain algorithms, such as back-projection,^11^ frequency-domain algorithms, such as Fourier transform-based methods,^12^ and iterative algorithms, such as model-based inversion.^13^ However, the computational complexity of these algorithms will face a significant challenge as the data size becomes significantly large due to an increase in both UST elements and imaging frames.

GPUs are highly parallel and efficient in executing large amounts of mathematical operations simultaneously, making them well-suited for various applications beyond graphics processing, including high-speed medical image reconstruction. Besides, GPUs are more cost-effective compared to other specialized hardware options, such as field-programmable gate arrays or application-specific integrated circuits (ASICs). Several research groups have developed GPU-accelerated methods for PACT reconstruction to address the computational challenges.^14^^–^^25^ However, there is a lack of detailed guidance on implementing GPU acceleration for various hardware configurations, especially for many research labs without expertise in GPU programming. Although CUDA C++ is preferred owing to its efficient parallel processing capabilities, its distinct programming model and syntax require a deep understanding of parallel computing and GPU architecture, making it challenging for most researchers not professionally trained in related computer science.

Currently, there have been some standardized attempts for PACT, such as IPASC,^26^ aim to provide standards for data formats, phantom properties, image quality characteristics, and clinical requirements; and graphical user interface applications, such as PATLAB.^27^ However, these attempts still lack efficient GPU image reconstruction algorithms. This is because of not only the difficulty in programming and integrating CUDA C++ programs but also challenging issues with environment configuration and platform compatibility. In contrast, our efforts are primarily focused on the development process for high-performance PACT reconstruction algorithms based on GPU, encompassing standardized raw data import, optimized reconstruction kernel functions, and the capability for multi-GPU usage. We provide a comprehensive framework for developing GPU-accelerated photoacoustic computed tomography, called GAPAT, that leverages widely used open-source parallel computing tools, such as Python multiprocessing-based parallelism, Taichi Lang for Python, CUDA, and possible other backends, which improves the convenience of programming these GPU reconstruction algorithms and ensures compatibility during integration, as well as facilitate the use of modern scientific computing libraries and AI tools, such as the three-dimensional (3D) image display and processing tool NAPARI^28^ (an interactive viewer for multidimensional images in Python), and the powerful image segmentation tool SAM^29^ (a zero-shot segmentation system proposed by Meta AI).

To leverage GPU capabilities, several programming tools have been developed. One of the main scientific computing tools used in GAPAT is the Taichi Lang for Python.^30^ Compared to CUDA C++, the Taichi allows developers to write GPU code using Python syntax, provide high-level abstractions, and facilitate seamless integration with existing Python workflows, making GPU programming much more friendly to developers, thus lowering the entry barrier for researchers with limited expertise in GPU programming and CUDA. Taichi is a cutting-edge programming language and framework designed for computer graphics, computational physics, and other high-performance numerical computation tasks,^31^^–^^33^ which is embedded within Python and uses just-in-time compilation architecture (such as LLVM) to translate Python source code into native instructions for GPU or CPU. A researcher familiar with Python can easily use Taichi and, by extension, our framework code.

This is especially true for PACT reconstruction, which frequently utilizes delay-and-sum (DAS) type algorithms and which primarily requires extensive indexing and vector summation operations, where vector summation’s most common parallel computing optimization technique is reduction. Taichi is capable of automatically implementing reduction optimizations via its compiler, achieving performance, such as highly optimized CUDA code, which is one of the main optimizations our framework provides. Besides, some other Python computation libraries, such as CuPy, can only implement more complex operations by writing CUDA code, making it challenging to be considered a high-performance programming library that is fully Python-fronted. The proposed GAPAT offers significant performance improvements for PACT image reconstruction, enabling faster analysis and real-time applications. By using the Taichi library, which abstracts away many of the complexities of parallel programming and enables developers to focus on writing their reconstruction algorithms, reconstruction programs can be feasibly adapted to run efficiently on a wide range of modern hardware platforms from CPU to GPU. In addition, the GAPAT can also extend image reconstruction algorithms to large-scale workstations and servers with multiple GPUs to use all computing resources of hardware.

## 2 Materials and Methods

**Fig. 1:**
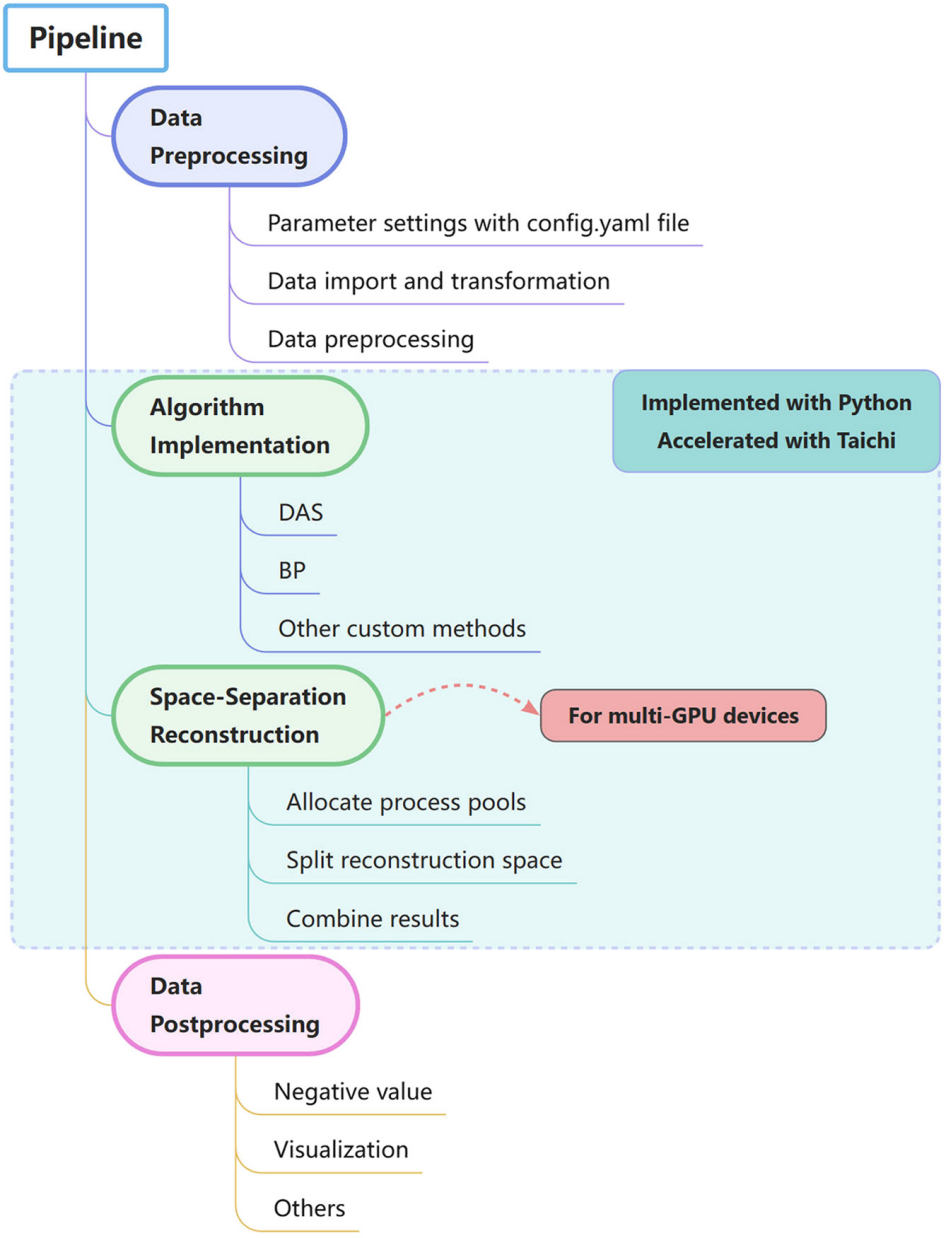
Flowchart of the pipeline of GAPAT.

Figure 1 describes the overall framework of GAPAT for developing GPU-accelerated PACT image reconstruction using open-source parallel computing tools. The framework consists of four main steps: data preprocessing, algorithm implementation, space-separation reconstruction, and data postprocessing. In the first step, we explain how to set the reconstruction system with config.yaml file and import data efficiently; in the second and third steps, we explain how to use Taichi and Python multiprocessing to perform parallel computing on different hardware platforms, especially multiple GPUs. In the last step, we perform some postprocessing to enhance the image quality and improve visualization of the target structure where you can use various Python image processing libraries, such as OpenCV, in our framework.

### 2.1 Data Preprocessing of GAPAT

#### 2.1.1 Reconstruction parameter settings

Currently, the PACT raw data primarily originate from two sources: simulation data and real experimental data stored in the data acquisition (DAQ) devices. Our framework uses a config.yaml file to extract the required parameters. YAML is a human-readable data serialization format. It is often used for configuration files and data exchange between languages with different data structures. Consequently, adjustments to parameters only necessitate changes to the config.yaml text file, facilitating parameter modification and source code exchange. In Table 1, the raw data are from a synthetic planar array by scanning a linear array of 256 elements at 1380 steps, equivalent to a large-scale matrix array with the size of 256×1380 elements.^34^

**Table 1:** Parameters in config.yaml file.

Parameters	Value	Explanation
vs	1500.0	Speed of sound, m/s
fs	40.0e+6	Sampling frequency, Hz
num_channels	256	Number of channels
num_steps	1380	Number of scan steps
num_times	2048	Number of time samples
res	0.20e-3	Spacing of the reconstruction area grid, m
data_path	!!str data	Path to the raw data
data_type	int16	Data type of raw data
device	gpu	Device that algorithms run on, cpu or gpu
num_devices	1	Number of devices that algorithms run on
…	…	…

#### 2.1.2 Data import and transformation

After reading the parameters from the config.yaml file, we proceed to import the raw data and complete the transforming process. Some parameters are necessary for the rapid importation of large-scale raw data, enabling us to utilize matrix operation functions in existing powerful numerical computation libraries to process multiple raw data files. This approach avoids the inefficiency of double loops and the repetitive operations often required when preloading a sub-file to obtain related data parameters in traditional methods. As a result, for example, GAPAT can read 1380 raw data files, each containing data collected by 256 detectors in a linear array with a length of 2048 time points, in just 2.2 s, from the *in vivo* experiments described in Sec. 3.2, with specific hardware parameters detailed in Sec. 3.1.

#### 2.1.3 Data preprocessing

Prior to the reconstruction process, the raw PA signals must be preprocessed. In our framework, data preprocessing steps may include: (1) bandpass filtering, (2) data resampling, and (3) specific preprocessing for algorithms. For instance, in the DAS algorithm provided within our framework, we set the first and last signal values of all original signals to zero, enabling efficient handling of out-of-bounds indices. Due to the efficient and compact data organization form adopted by the reshaped raw data, various data processing methods can be easily executed in parallel through matrix operations. This not only simplifies usage but also greatly enhances its extensibility.

### 2.2 Back-Projection Algorithm in GAPAT

There are two commonly used time-domain back-projection algorithms, the delay and sum (DAS) and filtered back-projection^11^ (FBP), for PACT. In our work, we implemented both algorithms in demonstration codes. The DAS is a relatively simple method, which is described as the following equation:

$$p_{0}(\overset{\to }{r})=\frac{\sum_{i=1}^{N}p_{i}({\overset{\to }{d}}_{i},t=\frac{\left|{\overset{\to }{d}}_{i}-\overset{\to }{r}\right|}{c}){\Delta \Omega }_{i}}{\sum_{i=1}^{N}{\Delta \Omega }_{i}}.$$ (1)

Noting that the ${\Delta \Omega }_{i}$ is the stereo angle of the i’th detector to the reconstruction point and that $\overset{\to }{d_{i}}$ is the position vector of i’th detector relative to the coordinate origin, $p_{0}(\vec{r})$ is the initial sound pressure at the reconstruction point with position vector $\vec{r}$, $p_{i}(t)$ represents the signal value of the i’th detector at delay t, and c is the speed of sound.

The FBP algorithm involves one more term of temporal derivative of PA signal, as follows:

$$p_{0}(\overset{\to }{r})=\frac{\sum_{i=1}^{N}[2(p_{i}({\overset{\to }{d}}_{i},t)-t\frac{\partial }{\partial t}p_{i}({\overset{\to }{d}}_{i},t)){]}_{t=\frac{\left|{\overset{\to }{d}}_{i}-\overset{\to }{r}\right|}{c}}{\Delta \Omega }_{i}}{\sum_{i=1}^{N}{\Delta \Omega }_{i}}.$$ (2)

In a large planar detection geometry, the normal direction of each UST is the same. Calculating the stereo angle, we can get that for a planar detector array, ignoring constant coefficients, the discrete equation becomes

$$\mathrm{DAS}:\;p_{0}(j)=\overset{N}{\underset{i=1}{\sum }}\frac{z_{j}}{l_{ij}^{3}}{\left[p_{i}(t)\right]}_{t=\frac{l_{ij}}{c}},$$ (3)

$$\mathrm{FBP}:\;p_{0}(j)=\overset{N}{\underset{i=1}{\sum }}\frac{z_{j}}{l_{ij}^{3}}{\left[p_{i}(t)-t\frac{\partial }{\partial t}p_{i}(t)\right]}_{t=\frac{l_{ij}}{c}}.$$ (4)

Here, $z_{j}$ represents the z-coordinate of the j’th reconstruction point and $l_{ij}$ represents the distance from the i’th detector to the j’th reconstruction point.

Currently, both algorithms have been implemented in our framework using parallel loops that iterate over the spatial grid points and the UST elements with Taichi Lang for Python. Specifically, we can implement a kernel in Python that executes the back-projection algorithm based on Taichi’s syntax. The Taichi compiler will then automatically apply various optimization techniques to generate highly optimized machine code for the target hardware. All one needs to do is to decorate the corresponding reconstruction functions as Taichi kernels. This flexibility in our framework allows the rapid development and testing of reconstruction algorithms while maintaining high computational performance.

### 2.3 Space-Separation Reconstruction Strategy of GAPAT

Despite Taichi kernels’ application, enabling multi-GPU PACT reconstruction remains challenging. This feature is not supported by Taichi kernels, and its implementation in CUDA C++ can be quite complicated. GAPAT offers a much easier solution based on Python multiprocessing, which creates asynchronous processes for each device (usually GPU), assigns subtasks, and integrates results upon completion. Then, we aim to divide the PACT reconstruction task into multiple subtasks for independent devices. In general, there are two approaches: partitioning either the detector array or the reconstruction space. The former faces issues with memory allocation in large or high-resolution spaces. Thus, we adopt the latter approach, dividing the reconstruction space into subspace regions at different depths, allowing efficient GPU allocation and memory management. This also enables depth-specific processing for improved reconstruction results. Specifically, taking a planar matrix detector array as an example, as shown in Algorithm 1, we divide the reconstruction space into subspace regions with different depths.

**Algorithm 1:** Space-separation reconstruction of FBP algorithm

**Input**: F: config file, N: number of devices
**Output**: I: reconstructed PACT image
1. Load configuration file F and set up all parameters
2. **Start** timing
3. Read all files in data directory into a signal matrix S
4. Get the detector locations into a matrix D
5. Initialize signal reconstruction matrix I
6. Define kernel function that performs FBP algorithm
7. Set up Taichi backend with CPU or CUDA
8. Create a pool of worker processes for all devices
9. **For** i=1 to N **do**
10. Apply kernel function for corresponding depth region into the $i^{th}$ process
11. Execute these kernel functions asynchronously
12. **End**
13. Concatenate results from all devices
14. Save signal reconstruction matrix I
15. **End** timing

### 2.4 Image Postprocessing

After the PACT reconstruction process, postprocessing is often performed to enhance the image quality, reduce noise, and improve visualization of results. GAPAT has provided some common postprocessing techniques for PACT, including:

#### 2.4.1 Negative value processing

Negative values in PACT reconstructed images can arise due to various factors, such as noise, artifacts, or limited view and limited bandwidth. Here are some of the methods GAPAT provides: (1) Absolute value: applying the absolute value function to the PACT reconstructed image. (2) Squaring: squaring the values in the reconstructed image. (3) Hilbert transform:^35^ the Hilbert transform is a linear operator that can be applied to PACT reconstructed images to obtain an analytic signal allowing for getting the envelope of the PA signal.

#### 2.4.2 Visualization

Enhance the visual representation of PACT images to facilitate interpretation and communication of the results. Visualization techniques currently in GAPAT mainly include volume rendering realized by OpenCV. The postprocessing techniques can be implemented using a variety of image processing and analysis software, such as MATLAB or some other Python libraries due to the powerful extensibility of our framework.

## 3 Results

Both simulated and experimental datasets were used to test and evaluate GAPAT. The simulated dataset was generated using a numerical phantom with solution of theoretical formula of photoacoustic wave function, while the experimental dataset was acquired from *in vivo* PACT imaging of a human forearm. Both datasets were tested with multiple hardware configurations, including multicore CPU, single GPU, and multiple GPU platforms. Depending on the available hardware resources, users can choose the most suitable configuration.

### 3.1 Simulation Results

**Fig. 2:**
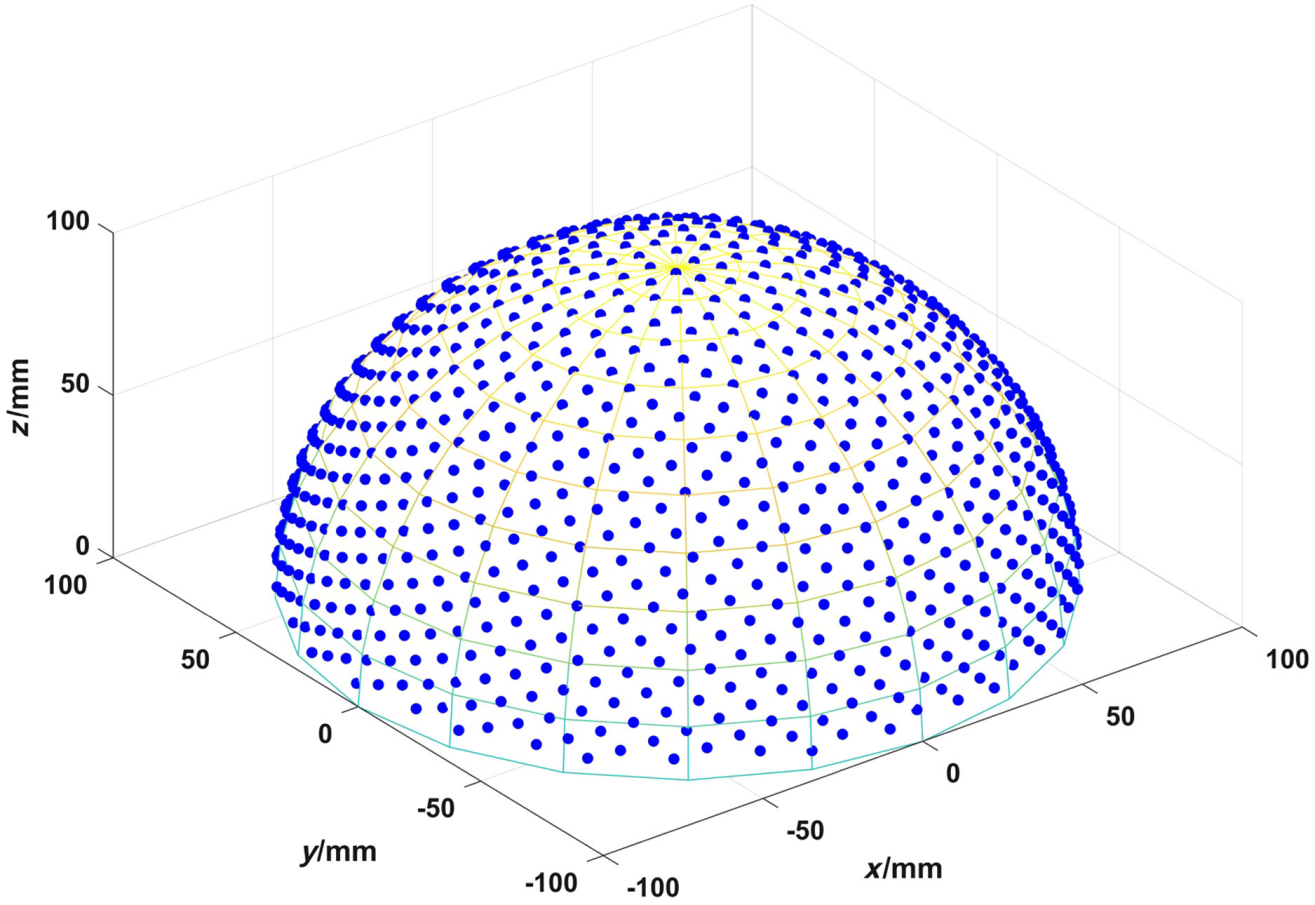
Detectors arranged in a Fibonacci array on a hemisphere.

In this study, we selected a PACT system using a hemispherical detector matrix array, which is an important setup for real-time 3D PACT. The hemispherical array has a radius of 100 mm with 1024 detectors. Considering the potential disadvantages of rotational symmetry on imaging, our simulation employed a spherical detector array arranged in a so-called “Fibonacci” pattern,^36^ as shown in Fig. 2.

Our simulation assumed each array element has an infinite wide bandwidth and infinitesimal point size. For the objects to be imaged, we chose a small sphere with a radius of 1 mm located at the center of the array, and the imaging field of view was set to a 5  mm×5  mm×5  mm spatial region near the center of the sphere. The sampling frequency in simulation is 40 MHz. Our hardware setup consists of an AMD Threadripper 3960X CPU, featuring 24 cores and 48 threads, 64 GB DDR4 memory, 2 NVIDIA GeForce RTX 3090 Ti 24 GB graphics cards. We chose the FBP algorithm to reconstruct the 3D image.

In this simulation, we compared the performance of three computational setups on various grid sizes: CPU parallel, single GPU, and dual GPU. Table 2 presents the time costs for each setup, respectively. It is essential to emphasize that while timing the execution of algorithms, we also include the time taken to read the original data from local files and save the reconstructed results back to the local storage for 3D PACT reconstructions that deal with a large volume of data. The observation that dual GPU configurations sometimes show less efficiency compared to a single GPU for smaller grid sizes is attributed to the overhead associated with managing parallel tasks across multiple GPUs. This overhead becomes negligible as the grid size increases. In summary, the dual GPU configuration offered the most efficient performance in terms of time cost, outpacing both the CPU parallel and single GPU setups.

**Table 2:** Simulation results with different grids.

Setup	Time cost (s) with 250×250×250 grids	Time cost (s) with 500×500×500 grids	Time cost (s) with 1000×1000×1000 grids
CPU parallel	6.83	45.1	426
Single GPU	2.30	3.33	13.7
Dual GPU	2.53	3.24	8.80

### 3.2 *In Vivo* Experimental Results

**Fig. 3:**
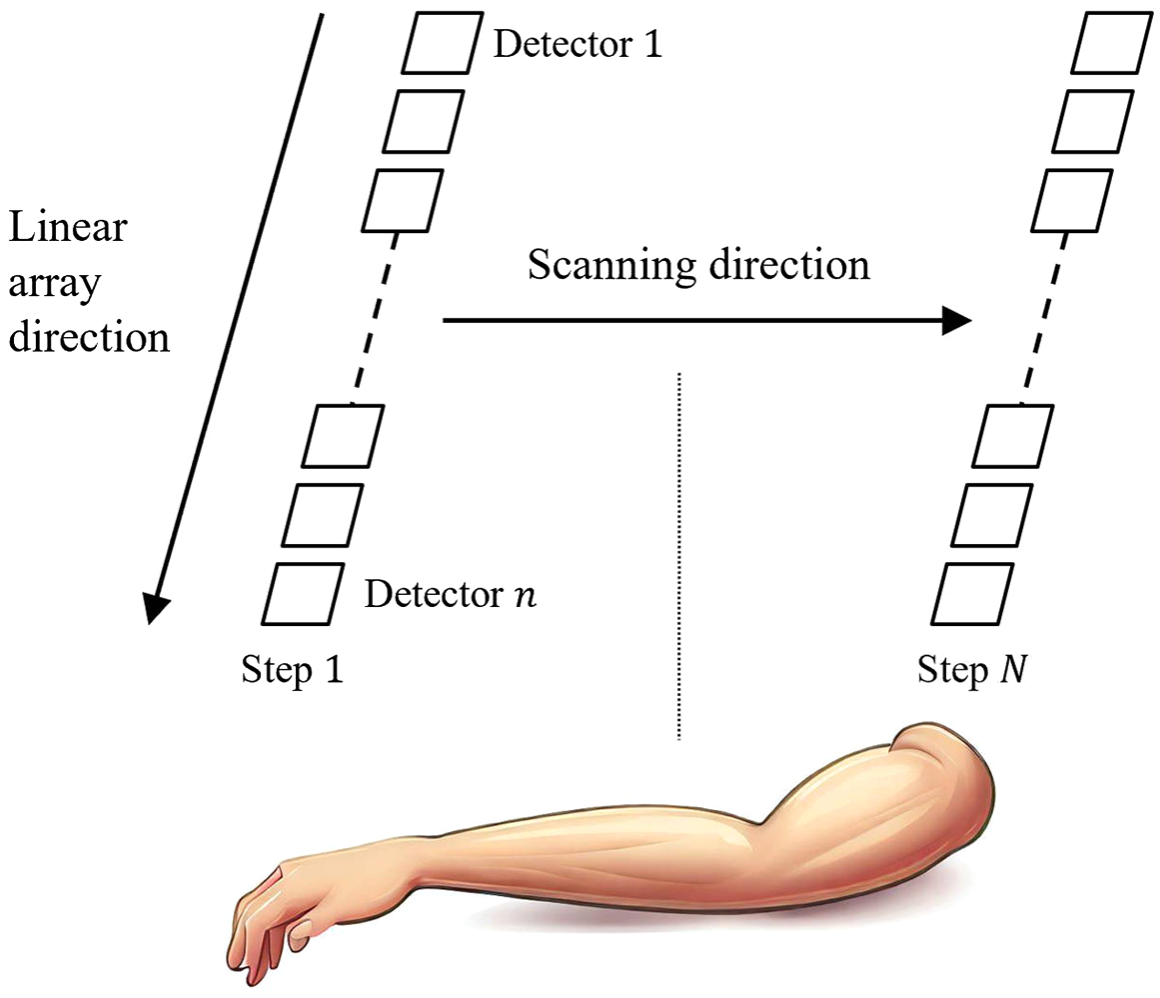
Schematic diagram of the scanning of linear array to form a synthetic planar array.

Our *in vivo* experiment data are from a PACT imaging of arm using a synthetic planar array.^34^^,^^37^ Our system employs a Q-switched Nd: YAG pulsed laser (LS-2137/2, LOTIS TII Ltd., Belarus) at 1064 nm for photoacoustic signal excitation. This laser produces pulses with a duration of ∼16  ns and has a repetition rate of 10 Hz. A non-focusing linear array consisting of 256 elements was used. The *in vivo* experiments utilized a data acquisition system (Marsonics DAQ, Tianjin Langyuan Technology Co., Ltd. China) at 40 MHz sampling rate. The raw data, in the form of int16, underwent a conversion process to match our framework’s input requirements, in the form of float32, ensuring compatibility and efficient processing. During imaging, we scanned the linear array above the arm over a total length of 138 mm at a scanning step of 0.1 mm, as shown in Fig. 3. In this case, an equivalent matrix array consists of ∼353  k elements (256×1380) is realized. At each detection each array element captures signal data containing 2048 time points.

In reconstructing the arm data of the following results, we use the DAS algorithm instead of the FBP algorithm for reconstruction to achieve higher robustness. Our framework, employing the DAS algorithm, incorporates the solid-angle factor consideration for planar arrays, as detailed in Eq. (3). In the case of planar arrays, the line connecting the detector and the reconstruction point often does not coincide with the normal vector of the detector array plane. It is necessary to multiply by the cosine of the angle difference to correct the signal during DAS reconstruction.

**Fig. 4:**
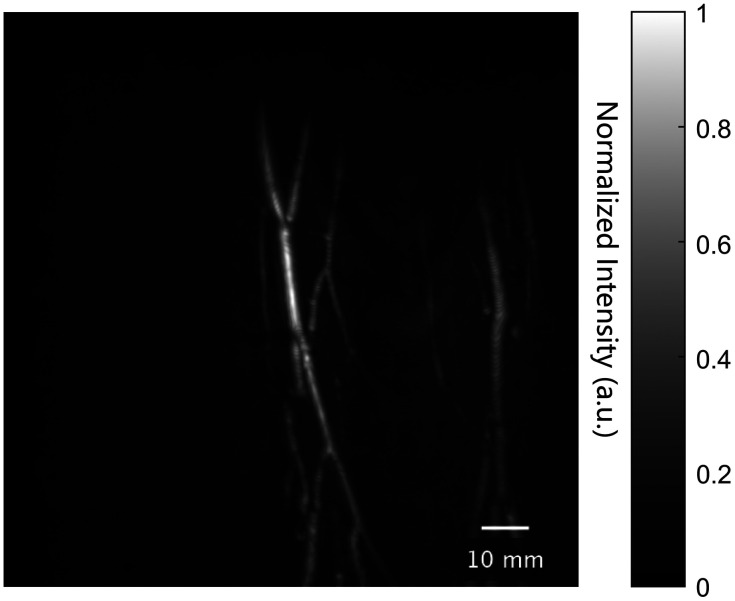
Reconstructed maximum intensity projection (MIP) image of the arm using GAPAT by 1200×1200×400 grids. Data that have been linearly normalized to fill the 0-1 interval.

We used the same hardware setup as that in the simulation study. Table 3 presents a comparison of the computational performance of three different setups—CPU parallel processing, single GPU, and dual GPU configurations—in PA imaging reconstructions using grid sizes of 240×240×80, 600×600×200, and 1200×1200×400, and Fig. 4 shows the MIP image reconstructed by single GPU and 1200×1200×400 grid.

**Table 3:** *In vivo* experimental results with different grids.

Setup	Time cost (s) with 240×240×80 grids	Time cost (s) with 600×600×200 grids	Time cost (s) with 1200×1200×400 grids
CPU parallel	3034 (0.84 h)	43,784 (12.2 h)	335,259 (93.1 h)
k-wave CPU	606.6	6044.7	44,690.4
k-wave GPU	18.7	768.4	Out of GPU memory
Single GPU	13.2	100.0	749.7
Dual GPU	10.8	55.0	385.1

The dual GPU setup still outperformed the other configurations. In summary, GAPAT can achieve an acceleration rate of ∼871 times when reconstructing extremely large-scale 3D PACT images on a dual-GPU platform compared to a 24-core workstation CPU, 116 times faster than k-Wave on a same CPU, and on this largest grids, the GPU-based algorithm in k-Wave directly exhausts the GPU memory and becomes inoperable, which highlights one of the major advantages of our method: more economical and efficient use of GPU memory. In addition to the above, we also compared the performance of GAPAT with a CUDA C++ coded program for this reconstruction task with a grid size of 600×600×200. The results indicate that, on this scale, the compiled CUDA C++ program takes 318 s (on a single GPU). As shown in Table 3, compared to the CUDA C++ program, our framework achieves a performance improvement of 3.2 times for a single GPU setup and 5.8 times for a dual GPU setup, respectively. The performance of GAPAT often surpasses that of manually coded CUDA C++ programs written by average developers. It is worth mentioning that GAPAT significantly reduces the difficulty of developing multi-GPU parallel programs compared to CUDA C++, which is crucial for 3D image reconstruction by large-scale PACT array systems.

Besides DAS, we also provide source code based on the FBP algorithm using GPU, the results of which can be found in the README.md file of our GitHub repository described in Sec. 4. According to our results, the optimized GPU-based FBP algorithm is only ∼5% slower on average compared to its DAS counterpart.

## 4 Discussion and Conclusion

Our above experiment results indicate that the GPU code of k-Wave is currently insufficient for large-scale 3D PACT reconstruction tasks due to its substantial memory requirements. Moreover, there has been a lack of updates and maintenance for these codes. The last update to k-Wave’s GPU code was made on February 28, 2020, and compatibility issues have been identified when utilizing the latest hardware. In addition, the recent published PACT toolbox PATATO^38^ (a Python photoacoustic tomography analysis toolkit) has garnered attention. However, since it employs Google’s JAX library for GPU acceleration, it only supports GPU on Linux platforms and faces the same challenges of underutilization of GPU memory compared to GAPAT. Given these issues, using PATATO may not be friendly for developers who wish to write their own GPU-accelerated new PACT reconstruction algorithms. Using the *in vivo* experimental data from Sec. 3.2 (with original data dimensions of 256×1380×2048), Table 4 still demonstrates the superiority of GAPAT.

**Table 4:** Comparative time efficiency of different systems and software configurations under different grid configurations.

Grid setup	Ours	PATATO				
	600*600*200	64*64*64	80*80*80	100*100*100	112*112*112	120*120*120
**Time (s)**	100.0	1.2	1.8	2.9	3.9	Out of memory
**Grid percentage**	7200%	26%	51%	100%	140%	173%
**Time percentage**	3448%	41%	62%	100%	134%	—
**Relative speed**	2.09	0.63	0.82	1.00	1.04	—

In practical applications, more scanning geometries exist beyond the spherical and planar geometries discussed in this work. To meet challenges in assigning normal directions of complex geometries within our framework, several strategies can be proposed: (1) user-defined normal direction: we could augment our framework to allow users to define the normal direction for each detector element manually. (2) Parametric representation: For certain complex geometries, a parametric representation might be used to describe the detector positions and orientations. (3) Hybrid approaches: for particularly intricate geometries, a hybrid approach might be necessary.

By directly interfacing with the data transmitted from the DAQ devices to the host memory, GAPAT can be adapted to facilitate real-time visualization of imaging results. This real-time capability necessitates certain modifications to the original data. Specifically, it requires a transformation and reordering process to ensure that each row of data distinctly represents the readings from an individual detector. It should be noted, however, that realizing this functionality often hinges upon the support provided by the DAQ manufacturer. Their proprietary SDKs (Software Development Kits) typically offer specialized methods for data manipulation and extraction.

In recent years, machine learning techniques have shown great potential for improving image reconstruction in various medical imaging modalities. Our framework can be extended to incorporate machine learning-based reconstruction algorithms, such as convolutional neural networks and recurrent neural networks, to further enhance the reconstruction performance and can keep the same hardware setup. By leveraging the massive parallelism of GPUs, our framework can efficiently implement machine learning-based algorithms, allowing for faster and more accurate PACT image reconstruction.

To facilitate the adaptation of our framework by the research community, we release the source code and documents used in this work under an open-source license. The code of a demo version of GAPAT is publicly accessible from the following GitHub repository: https://github.com/ddffwyb/GAPAT. It provides installation and usage instructions, as well as a set of sample data for execution. This will enable researchers to customize the framework to meet their specific needs, as well as to contribute improvements and new features that can benefit the entire community.

In conclusion, our comprehensive framework for GPU-accelerated PACT reconstruction aims to address computational challenges associated with PACT image reconstruction, offering a flexible and high-performance solution that can be tailored to different hardware configurations. By promoting faster analysis and applications, our framework contributes to the advancement of PACT imaging and its broader adoption in science research and clinical settings.

## Data Availability

The code and data of a demo version of GAPAT are publicly accessible from the following GitHub repository: https://github.com/ddffwyb/GAPAT. It provides a set of sample data for execution.

## References

[1] Dean-Ben X. L. et al., “Advanced optoacoustic methods for multiscale imaging of *in vivo* dynamics,” Chem. Soc. Rev. 46, 2158–2198 (2017). CSRVBR 0306-0012 10.1039/c6cs00765a 28276544 PMC5460636

[2] Wang L. V. Yao J., “A practical guide to photoacoustic tomography in the life sciences,” Nat. Methods 13, 627–638 (2016).1548-7091 10.1038/nmeth.3925 27467726 PMC4980387

[3] Xu M. Wang L. V., “Photoacoustic imaging in biomedicine,” Rev. Sci. Instrum. 77, 041101 (2006). RSINAK 0034-6748 10.1063/1.2195024

[4] Li C. Wang L. V., “Photoacoustic tomography and sensing in biomedicine,” Phys. Med. Biol. 54, R59–R97 (2009). PHMBA7 0031-9155 10.1088/0031-9155/54/19/R01 19724102 PMC2872141

[5] Wang L. V., “Prospects of photoacoustic tomography,” Med. Phys. 35, 5758–5767 (2008). MPHYA6 0094-2405 10.1118/1.3013698 19175133 PMC2647010

[r6] Xia J. Yao J. Wang L. V., “Photoacoustic tomography: principles and advances,” Electromagn. Waves 147, 1–22 (2014). 10.2528/PIER14032303 PMC4311576 25642127

[r7] Tian C. et al., “Spatial resolution in photoacoustic computed tomography,” Rep. Prog. Phys. 84, 036701 (2021). RPPHAG 0034-4885 10.1088/1361-6633/abdab9 33434890

[8] Na S. Wang L. V., “Photoacoustic computed tomography for functional human brain imaging,” Biomed. Opt. Express 12, 4056–4083 (2021). BOEICL 2156-7085 10.1364/BOE.423707 34457399 PMC8367226

[9] Fatima A. et al., “Review of cost reduction methods in photoacoustic computed tomography,” Photoacoustics 15, 100137 (2019). 10.1016/j.pacs.2019.100137 31428558 PMC6693691

[10] Na S. et al., “Massively parallel functional photoacoustic computed tomography of the human brain,” Nat. Biomed. Eng. 6, 584–592 (2022). 10.1038/s41551-021-00735-8 34059809 PMC8630100

[11] Xu M. Wang L. V., “Universal back-projection algorithm for photoacoustic computed tomography,” Phys. Rev. E 71, 016706 (2005). 10.1103/PhysRevE.71.016706 15697763

[12] Xu Y. Feng D. Wang L. V., “Exact frequency-domain reconstruction for thermoacoustic tomography. I. Planar geometry,” IEEE Trans. Med. Imaging 21, 823–828 (2002). ITMID4 0278-0062 10.1109/TMI.2002.801172 12374319

[13] Dean-Ben X. L. et al., “Accurate model-based reconstruction algorithm for three-dimensional optoacoustic tomography,” IEEE Trans. Med. Imaging 31, 1922–1928 (2012). ITMID4 0278-0062 10.1109/TMI.2012.2208471 23033065

[14] Peng K. et al., “Three-dimensional photoacoustic tomography based on graphics-processing-unit-accelerated finite element method,” Appl. Opt. 52, 8270–8279 (2013). APOPAI 0003-6935 10.1364/AO.52.008270 24513828

[r15] Yuan J. et al., “Real-time photoacoustic and ultrasound dual-modality imaging system facilitated with graphics processing unit and code parallel optimization,” J. Biomed. Opt. 18, 086001 (2013). JBOPFO 1083-3668 10.1117/1.JBO.18.8.086001 23907277 PMC3733419

[r16] Shan T. et al., “GPU-based acceleration and mesh optimization of finite-element-method-based quantitative photoacoustic tomography: a step towards clinical applications,” Appl. Opt. 56, 4426–4432 (2017). APOPAI 0003-6935 10.1364/AO.56.004426 29047873

[r17] Vu T. Wang Y. Xia J., “Optimizing photoacoustic image reconstruction using cross-platform parallel computation,” Vis. Comput. Ind. Biomed. 1, 2 (2018). 10.1186/s42492-018-0002-5 PMC7089714 32226922

[r18] Miri Rostami S. R. et al., “GPU-accelerated double-stage delay-multiply-and-sum algorithm for fast photoacoustic tomography using LED excitation and linear arrays,” Ultrasonics Imaging 41, 301–316 (2019). 10.1177/0161734619862488 31322057

[r19] Jeon S. et al., “Real-time delay-multiply-and-sum beamforming with coherence factor for *in vivo* clinical photoacoustic imaging of humans,” Photoacoustics 15, 100136 (2019). 10.1016/j.pacs.2019.100136 31467842 PMC6710719

[r20] Zhang Y. Wang L., “Video-rate ring-array ultrasound and photoacoustic tomography,” IEEE Trans. Med. Imaging 39, 4369–4375 (2020). ITMID4 0278-0062 10.1109/TMI.2020.3017815 32813650

[r21] Gonzalez E. A. Bell M. A. L., “GPU implementation of photoacoustic short-lag spatial coherence imaging for improved image-guided interventions,” J. Biomed. Opt. 25, 077002 (2020). JBOPFO 1083-3668 10.1117/1.JBO.25.7.077002 32713168 PMC7381831

[r22] Gao M. et al., “Graphics processing unit accelerating compressed sensing photoacoustic computed tomography with total variation,” Appl. Opt. 59, 712–719 (2020). APOPAI 0003-6935 10.1364/AO.378466 32225199

[r23] Lin L. et al., “High-speed three-dimensional photoacoustic computed tomography for preclinical research and clinical translation,” Nat. Commun. 12, 882 (2021). NCAOBW 2041-1723 10.1038/s41467-021-21232-1 33563996 PMC7873071

[r24] Paul S. et al., “Simplified-delay-multiply-and-sum-based promising beamformer for real-time photoacoustic imaging,” IEEE Trans. Instrum. Meas. 71, 4006509 (2022). IEIMAO 0018-9456 10.1109/TIM.2022.3187734

[25] Sun Y. Jiang H., “Enhancing finite element-based photoacoustic tomography by localized reconstruction method,” Photonics 9, 337 (2022). 10.3390/photonics9050337

[26] Bohndiek S. E. et al., “IPASC: a community-driven consensus-based initiative towards standardisation in photoacoustic imaging,” in IEEE Int. Ultrasonics Symp. (IUS) (2020). 10.1109/IUS46767.2020.9251362

[27] Omidi P. et al., “PATLAB: a graphical computational software package for photoacoustic computed tomography research,” Photoacoustics 28, 100404 (2022). 10.1016/j.pacs.2022.100404 36185542 PMC9520073

[28] Chiu C. Clack N., and The Napari Community, “Napari: a Python multi-dimensional image viewer platform for the research community,” Microsc. Microanal. 28, 1576–1577 (2022). MIMIF7 1431-9276 10.1017/S1431927622006328

[29] Kirillov A. et al., “Segment anything,” Cornell University Library, Ithaca, arXiv.org (2023).

[30] Hu Y. et al., “Taichi: a language for high-performance computation on spatially sparse data structures,” ACM Trans. Graphics 38, 1–16 (2019). ATGRDF 0730-0301 10.1145/3355089.3356506

[31] Hu Y. et al., “DiffTaichi: differentiable programming for physical simulation,” 10.48550/arXiv.1910.00935, 1910.00935 (2019).

[r32] Mukherjee D. et al., “Fast multipole methods for N-body simulations of collisional star systems,” Astrophys. J. 916, 9 (2021). ASJOAB 0004-637X 10.3847/1538-4357/ac03b2

[33] Yang J. et al., “Taichi-LBM3D: a single-phase and multiphase lattice Boltzmann solver on cross-platform multicore CPU/GPUs,” Fluids 7, 270 (2022). 10.3390/fluids7080270

[34] Li S. et al., “Photoacoustic imaging of peripheral vessels in extremities by large-scale synthetic matrix array,” J. Biomed. Opt. 29, S11519 (2024). JBOPFO 1083-3668 10.1117/1.JBO.29.S1.S11519 38259508 PMC10800540

[35] Li G. et al., “Multiview Hilbert transformation for full-view photoacoustic computed tomography using a linear array,” J. Biomed. Opt. 20, 066010 (2015). JBOPFO 1083-3668 10.1117/1.JBO.20.6.066010 26112369 PMC4481023

[36] Swinbank R. James Purser R., “Fibonacci grids: a novel approach to global modelling,” Q. J. R. Meteorol. Soc. 132, 1769–1793 (2006). QJRMAM 0035-9009 10.1256/qj.05.227

[37] Zhang G. et al., “Developing a photoacoustic whole-breast imaging system based on the synthetic matrix array,” Front. Phys. 8, 529 (2020). 10.3389/fphy.2020.600589

[38] Else T. R. et al., “PATATO: a Python photoacoustic tomography analysis toolkit,” J. Open Source Software 9(93), 5686 (2024). 10.21105/joss.05686

